# Cognitive and neuroanatomical assessment of alexia and agraphia in Japanese: implications for the European languages

**DOI:** 10.3389/fnhum.2025.1664803

**Published:** 2025-09-04

**Authors:** Yasuhisa Sakurai

**Affiliations:** ^1^Department of Health Care, Mitsui Memorial Hospital, Tokyo, Japan; ^2^Takuma Neurology Clinic, Tokyo, Japan

**Keywords:** kanji, kana, dual-route hypothesis, pure alexia, alexia with agraphia

## Abstract

The Japanese language has a unique writing system that consists of *kanji* (morphograms, derived from Chinese characters) and *kana* (phonograms, a simplified form of kanji representing syllables). A kanji character has two distinct ways of reading: *on*-reading (Chinese-style pronunciation) and *kun*-reading (native Japanese pronunciation). Some kanji words have irregular *kun*-reading called *jukujikun*. Furthermore, kana characters have two script forms: *hiragana* (cursive form) and *katakana* (square form), each of which is used for different purposes. Because of these features, Japanese individuals with alexia and agraphia show characteristic symptoms. Lesion-to-symptom analyses and functional imaging studies developed beginning in the 1970s have reported the following findings: (1) kanji–kana dissociation in alexia/agraphia: pure alexia for kanji or kana, lexical agraphia for kanji, and phonological agraphia for kana; (2) *on-kun* dissociation in alexia: predominant *kun*-reading and *jukujikun* reading impairment in semantic dementia and selective *on*-reading impairment in the extensive posterior middle temporal gyrus lesion; and (3) allographic agraphia between *hiragana* and *katakana*.

## 1 Introduction

Due to the writing systems used for the Japanese language, reading and writing impairments in Japanese people have led to dissociative differences in performance between *kanji* (morphograms) and *kana* (phonograms). Early case studies reported kanji- or kana-predominant alexia or agraphia in various aphasia syndromes. These included kana agraphia in progressive nonfluent aphasia (Watanabe, 1893) and Broca’s aphasia (Miura and Okada, 1901). In contrast, Imura (1943) first reported kanji alexia and agraphia in Gogi (word-meaning) aphasia, a linguistic manifestation of semantic dementia or semantic variant primary progressive aphasia (Gorno-Tempini et al., 2011). Lesion-symptom studies have advanced since the 1970s, with the development of brain computed tomography and later magnetic resonance imaging. Sasanuma (1975) investigated reading and writing impairment in various types of Japanese aphasics and reported that errors in kana reading and writing were more frequent than kanji errors were in individuals with Broca’s aphasia, Wernicke’s aphasia, and conduction aphasia. However, later studies, including that of Sasanuma, suggested that the kana-predominant alexia or agraphia in aphasia syndrome is not always observed (Hamanaka et al., 1980; Sasanuma, 1980). Conversely, kanji-specific or kana-specific cases of nonaphasic alexia or agraphia have been reported. In particular, one study reported alexia with agraphia for kanji without accompanying aphasia from a lesion of the left posterior inferior temporal cortex (PITC) (Iwata, 1984). Furthermore, pure alexia for kana was reported following damage to the posterior occipital gyri (Sakurai et al., 2001a).

Paradis et al. (1985) conducted a review of cases of alexia with agraphia published from 1901 to 1983. For a historical review of this kanji–kana dissociation, see Sakurai (2019). A recent review of kanji/kana alexia and agraphia has also been published (Suzuki, 2022).

In this article, I discuss the cognitive neuropsychological aspects and anatomical substrates of alexia and agraphia, reviewing the lesion-to-symptom analyses and functional imaging studies conducted over the past 40 years with special reference to the Japanese language. This article aims to elucidate the various manifestations of alexia and agraphia in Japan and show how these features are unified in the understanding of the language.

## 2 Brief description of the Japanese writing system

First, the fundamental features of the Japanese language are described. Japanese is a unique language in that it has two distinct writing systems: kanji and kana. Kanji, which means “Chinese characters,” was originally transported from China to Japan in the fifth century or earlier (Yamadori, 2007).

**Figure 1:**
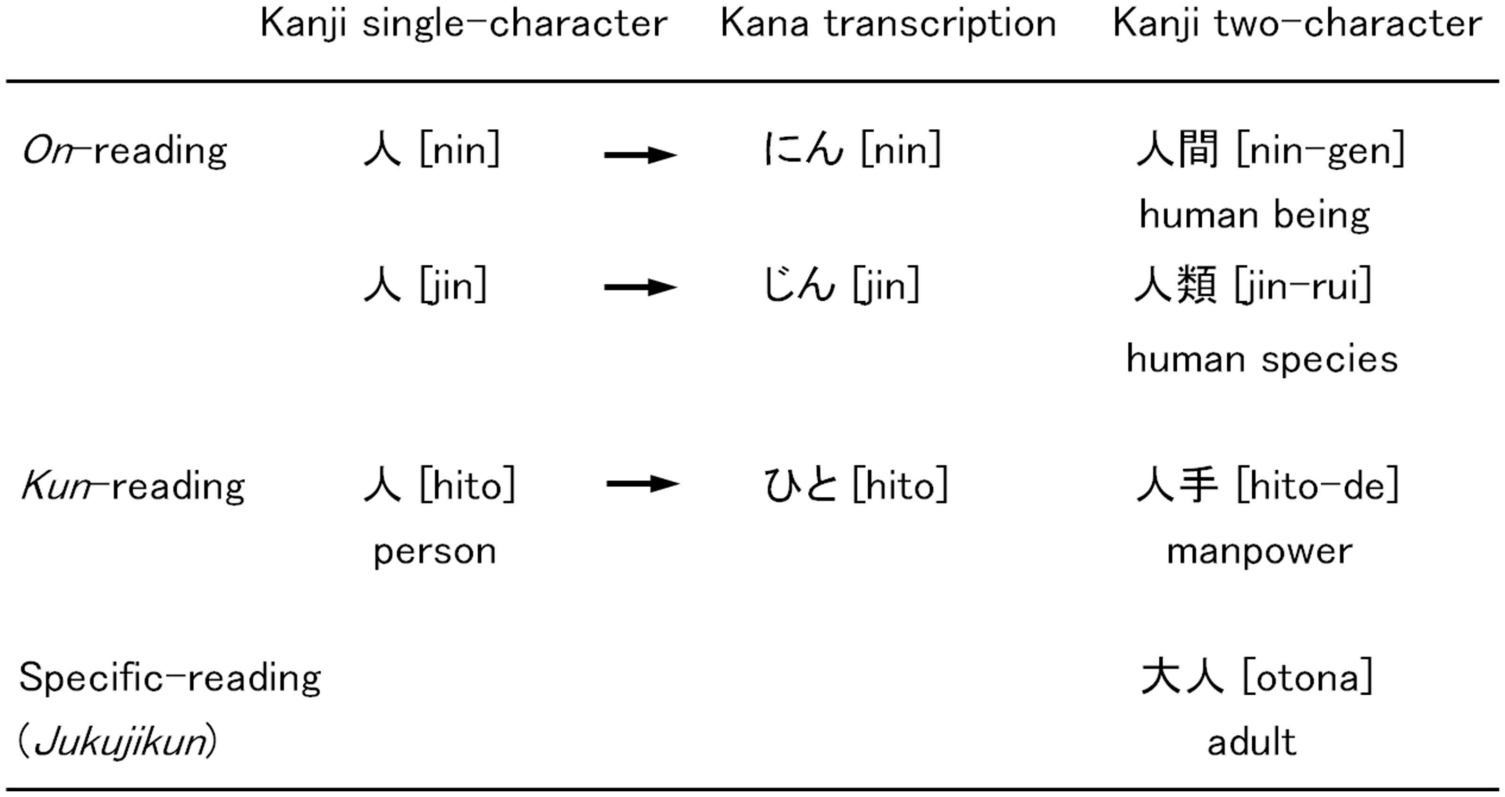
Examples of single-character and two-character kanji words. The word 人 ([hito], person) has two *on* (phonetic)-readings (Chinese-style pronunciation) depending on the era of transport from China and one *kun* (semantic)-reading (native Japanese pronunciation) attached to its meaning. *On*-reading or *kun*-reading of the single character is used to read two-character kanji words, as in *on–on*-reading (人間) or *kun-kun*-reading (人手). Specific-reading called *jukujikun* assigns Chinese characters to the native Japanese pronunciation. As a result, two-character kanji words with specific-reading are not pronounced using the *on*-reading or *kun*-reading of the original single-character kanji.

Many kanji characters are orthographically complicated and require multiple-stroke sequences to write. A kanji character has multiple *on*-readings (Chinese-style pronunciation), depending on the era when the character was transported, and *kun*-readings (native Japanese pronunciation), which are essentially the Japanese translation of the Chinese characters (Figure 1).

*Kana*, which means “temporary name,” was developed by simplifying kanji characters to represent syllables. The kana syllabary contains 46 basic syllables or morae that consist of consonant–vowel syllables and a few consonant-only and vowel-only syllables of short duration. Kana resembles the Roman alphabet in that both are phonographic systems, with each character used to build a word. However, a fundamental difference is that kana uses symbols for syllables, whereas the Roman alphabet uses symbols for individual phonemes (Kess and Miyamoto, 1999).

There are two types of kana script: *hiragana* (a cursive form of kana) and *katakana* (a square form of kana). *Hiragana* is derived from the running style of a kanji character and is used for grammatical morphemes and the transcription of kanji words. In contrast, *katakana* is derived from parts of a kanji character and is primarily used for words that are borrowed from other languages, that is, loan words. It is also used for representing names of foreign people, biological species, onomatopoeic words that sound similar to the actual noises, and for stylistic emphasis (Igarashi, 2007). Similar to English upper- and lowercase letters, *hiragana* and *katakana* can be considered allographs in the sense that both have the same phonetic value and 10 characters are orthographically analogous (e.g., *hiragana* か [ka] and *katakana* カ [ka]) (Sakurai et al., 2010).

A word is composed of several combinations of kanji or kana characters. A kanji character itself can be both a word and a grapheme. Two-character kanji words comprise the majority of kanji vocabulary. In a standard Japanese kanji dictionary (Kimura and Kurosawa, 1996), 93.5% of words contain two characters, 3.9% three characters, 2.5% four characters, and 0.07% five characters in all kanji (two- to five-character) words excluding proper nouns. They are predominantly read with *on–on* reading (95.8% of all two-character kanji words) (e.g., 朝食 [chou-shyoku] “breakfast”), *kun-kun* reading (3.1%) (e.g., 建物 [tate-mono] “building”), and, less frequently, *on-kun* (0.5%) and *kun-on* readings (0.6%). Some two-character kanji are read with specific-reading, called *jukujikun*. The specific-reading is an irregular *kun*-reading (Kess and Miyamoto, 1999) that assigns semantically related Chinese characters to native Japanese words (Kindaichi, 1988), for example, 大人 [otona] “adult,” where 大 [ookii] means “large” and 人 [hito] means “person” (Figure 1).

## 3 Cognitive model of reading, writing, and naming

**Figure 2:**
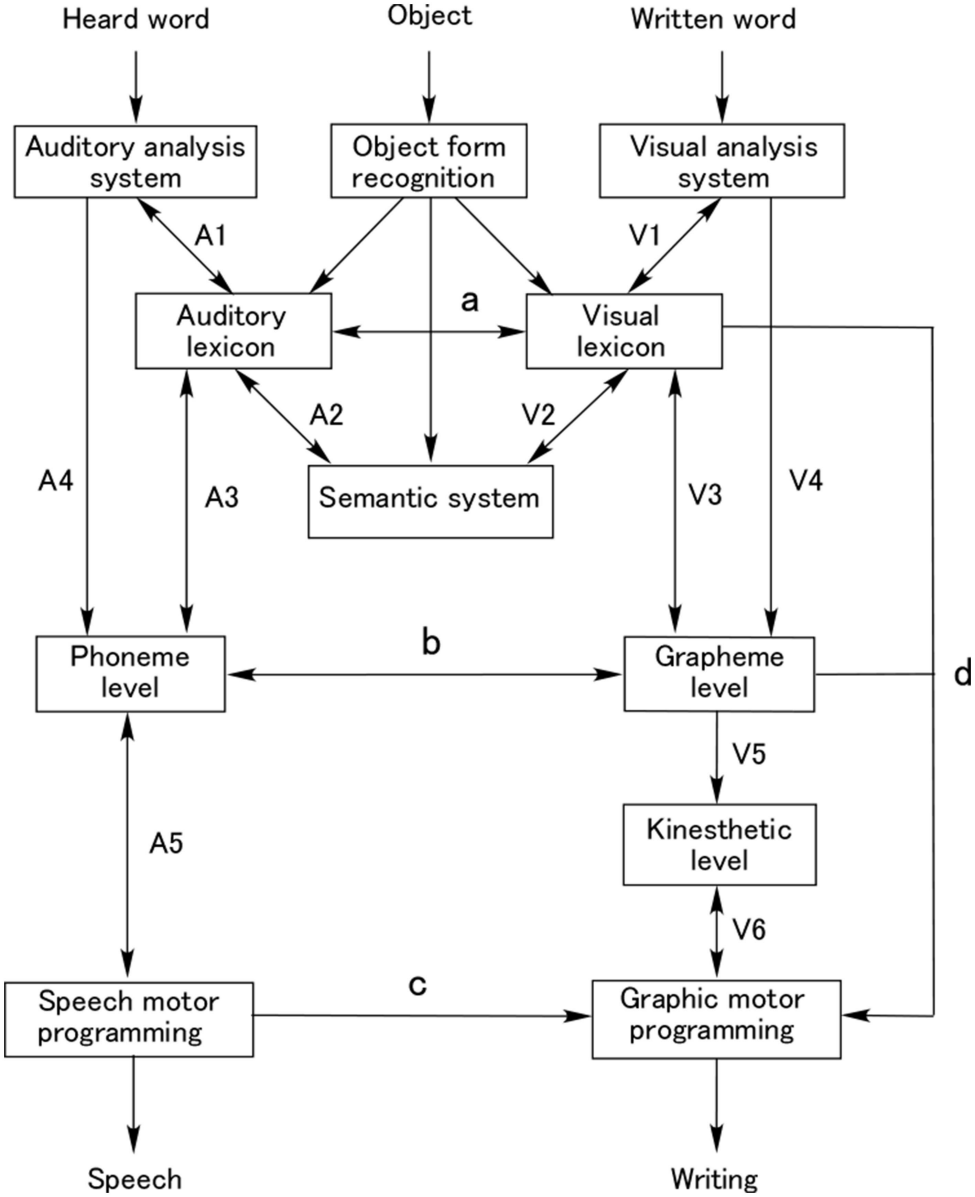
A composite model of reading, writing, and object naming. The process of reading aloud consists of the lexical–semantic route (V1 → V2 → A2 → A3 → A5), the direct lexical route without semantics (V1 → a → A3 → A5), and the nonlexical grapheme–phoneme conversion route (V4 → b → A5). The process of writing from dictation consists of the lexical–semantic route (A1 → A2 → V2 → V3 → V5 → V6), the direct lexical route without semantics (A1 → a → V3 → V5 → V6), and the nonlexical phoneme–grapheme conversion route (A4 → b → V5 → V6). Writing requires two other processes: the direct phonological route (A4 [or A1 → A3] → A5 → c) and the direct orthographic route (A1 → a → d, or A4 → b → d). Object naming proceeds from visual recognition directly to the auditory lexicon, visual lexicon, and semantic knowledge.

To explain the kanji–kana dissociation, several cognitive models, particularly for reading, have been proposed (for review, see Sato, 2015). Early models adopted the dual-route hypothesis and distinguished the lexical–semantic route for kanji reading and the nonlexical grapheme–phoneme conversion route for kana reading only (Sasanuma, 1987) or both kanji and kana reading (Paradis et al., 1985). Later, researchers used the triangle model (Plaut et al., 1996) to explain the cases of kanji–kana difference, that is, surface dyslexia (Patterson et al., 1995) and phonological dyslexia (Patterson, 1996). Furthermore, composite models including reading and writing were developed. Figure 2 shows the author’s cognitive model of reading, writing, and naming, based on those of Patterson and Shewell (1987), Ellis and Young (1997), Whitworth et al. (2014), and Beeson and Rapcsak (2010). In this model, the lexical–semantic route (V1 → V2 → A2 → A3) and phonological (nonlexical) route (V4 → b) for reading, and the lexical–semantic route (A1 → A2 → V2 → V3) and phonological (nonlexical) route (A4 → b) for writing were illustrated.

The model differs from previous models mainly with regard to the following points:

1. The grapheme–phoneme conversion in reading and the phoneme–grapheme conversion in writing are processed in the same module (b in Figure 2), because these two processes may be anatomically localized in the same area.
2. There is no distinction between input and output lexicon: the auditory input and output lexicons are unitary as are the visual input and output lexicons. This is because lexical knowledge is assumed to be localized in a cortical area.
3. The lexical–semantic route has a bypass pathway named the “direct route” between the auditory lexicon and visual lexicon (a). This route is used in reading or writing without referring to semantic knowledge.

Kana are read in both the phonological and lexical–semantic routes, whereas kanji are read exclusively in the lexical–semantic route (described in the anatomically constrained model).

## 4 Classification of alexia and agraphia

Alexia is an inability to read letters, words, or sentences despite preservation of visual acuity and the visual field. In contrast, agraphia is a writing or spelling disorder, despite preservation of motor–sensory function necessary for writing. Although both alexia and agraphia can be accompanied by aphasia, they can appear without accompanying aphasia. The cognitive neuropsychological classification includes both aphasic and nonaphasic alexia/agraphia, whereas the neuroanatomical classification distinguishes aphasic alexia/agraphia and nonaphasic alexia/agraphia.

### 4.1 Cognitive neuropsychological classification

Shallice and Warrington (1986) first proposes the distinction between peripheral and central dyslexia. Peripheral dyslexia occurs at the level prior to visual recognition of words, whereas central dyslexia occurs at the level from visual word recognition to phonology or semantics.

Central dyslexia includes phonological dyslexia (the selective impairment of nonword reading), surface dyslexia (the selective impairment of irregular word reading), and deep dyslexia (phonological dyslexia plus semantic paralexia) (Table 1). Different manifestations in these dyslexias are presented by kanji and kana reading (Table 2).

**Table 1:** Cognitive neuropsychological classification of alexia.

I. Central dyslexia
Phonological dyslexia
Surface dyslexia
Deep dyslexia
II. Peripheral dyslexia
Letter-by-letter reading
Neglect dyslexia
Attentional dyslexia

Based on Coslett (2011).

**Table 2:** Reading and writing errors in central dyslexia and agraphia in Japanese patients.

Types of alexia/agraphia	Nonword	Regular word	Irregular word
Alexia			
Phonological dyslexia	I	P	P
Surface dyslexia	P (kana)	I (kanji)	I (kanji)
Deep dyslexia	I	I (semantic error)	I (semantic error)
Agraphia			
Phonological agraphia	I (kana)	P	P
Lexical agraphia surface agraphia	P (kana)	I (kanji)	I (kanji)
Deep agraphia	I (kana)	I (semantic error; kanji)	I (semantic error; kanji)

I, impaired; P, preserved.

Similarly, agraphia is divided into central (linguistic) agraphia and peripheral (motor) agraphia (Ellis and Young, 1997). Central agraphia refers to impaired access to the visual images required for writing, whereas peripheral agraphia refers to the interruption of converting from a visual image to a kinesthetic pattern of words.

Central agraphia includes phonological agraphia (the selective impairment of nonword writing), lexical/surface agraphia (the selective impairment of irregular word writing), and deep agraphia (phonological agraphia plus semantic paragraphia) (Table 3). Some authors identified lexical agraphia with surface agraphia (Ullrich and Roeltgen, 2011); others subdivided surface agraphia into a semantic type and orthographic type (Beeson and Rapcsak, 2015). However, lexical agraphia should be distinguished from surface agraphia in that lexical agraphia results from impairment of the orthographic lexicon or its connection to auditory input/output, whereas surface agraphia is caused by impairment of the semantic memory. This view is supported by the fact that regularization (phonologically plausible kanji writing, described later, e.g., 新文 [shinbun], a pseudohomophonous new word for 新聞 [shinbun], “newspaper”) errors are observed in the surface agraphia of semantic dementia (Sakurai et al., 2006a) but not in lexical agraphia or kanji agraphia resulting from focal lesions of the temporal and parietal lobes (Sakurai et al., 2000b; Sakurai et al., 2008a).

**Table 3:** Cognitive neuropsychological classification of agraphia.

I. Central (linguistic) agraphia
Phonological agraphia
Lexical (orthographic) agraphia
Surface agraphia
Deep agraphia
II. Peripheral (motor) agraphia
Apraxic agraphia
Allographic agraphia
cf. Somesthetic dysgraphia

Based on Ullrich and Roeltgen (2011) and Beeson and Rapcsak (2010).

On the other hand, peripheral agraphia includes apraxic agraphia (the inability to realize well-formed graphemes despite the preserved visual images of words) and allographic agraphia (confusion between uppercase and lowercase) (Table 3). Depending on the word type (nonword, regular word, and irregular word), kanji and kana writing also show dissociable differences (Table 2).

Specifically, we previously reported cases of somesthetic dysgraphia (Sakurai et al., 2007; Mitani and Sakurai, 2024). This agraphia type is characterized by illegible grapheme formation due to impaired deep and combined sensation (stereognosis, two-point discrimination, graphesthesia, etc.), resulting in insufficient proprioceptive feedback to the motor programming. Somesthetic dysgraphia may be classified as peripheral agraphia (Table 3).

Kana alexia/agraphia resulting from disrupted grapheme–phoneme conversion is considered phonological dyslexia/agraphia, because greater impairment is expected for kana nonword reading/writing than for kana word reading/writing. In contrast, kanji agraphia resulting from impaired character recall is considered lexical agraphia, as kanji words with irregular reading are expected to be more difficult to read or write than those with regular reading.

### 4.2 Neuroanatomical classification

We proposed a classification of alexia/agraphia in accordance with the results of lesion studies (Sakurai et al., 2010; Table 4). As previously described, alexia and agraphia are first divided into aphasic and nonaphasic types. Nonaphasic alexia/agraphia is further divided into a primary type (i.e., caused by lesions related to reading and writing) and a secondary type (i.e., caused by lesions unrelated to language that affect reading or writing performance). In primary nonaphasic alexia/agraphia, kanji or kana selectivity is added to each of the alexia/agraphia types (second and third columns in Table 4).

**Table 4:** Neuroanatomical classification of alexia/agraphia.

Types of alexia/agraphia	Alexia	Agraphia
I. Aphasic alexia/agraphia		
II. Primary nonaphasic alexia/agraphia		
1. Pure alexia		
A. Splenium type: medial occipital gyri (or optic radiation, lateral geniculate body) + splenium (or callosal radiation, forceps major)	kanji >= kana^a^	
B. Nonsplenium type		
Fusiform type: mid-fusiform gyrus (BA 37)	kanji > kana	
Posterior occipital type: post-fusiform/inferior occipital gyri (BA 18/19)	kana	
2. Alexia with agraphia		
A. Angular type: angular/lateral occipital gyri (BA 39/19)	kana	kanji
B. Posterior inferior temporal type: mid-fusiform/inferior temporal gyri (BA 37)	kanji	kanji
3. Pure agraphia		
A. Posterior middle temporal gyrus (BA 21/37)		kanji
B. Angular gyrus (BA 39)		kanji
C. Supramarginal gyrus (BA 40)		kana
D. Superior parietal lobule (intraparietal sulcus, BA 7)		kanji > kana
E. Posterior middle frontal gyrus (BA 6)		kanji > < kana^b^
III. Secondary nonaphasic alexia/agraphia		
1. Alexia		
A. Hemianopic alexia		
B. Neglect dyslexia		
C. Callosal alexia		
D. Thalamic alexia		
2. Agraphia		
A. Constructional agraphia		
B. Neglect dysgraphia (spatial agraphia)		
C. Callosal agraphia		
D. Thalamic agraphia		

BA, Brodmann area. Revised with permission from Sakurai et al. (2010).

a Reading is more impaired in kanji than in kana or is equally impaired.

b Writing is more (or less) impaired in kanji than in kana.

The classification has several points that have not been highlighted before. These points are discussed in the following session in accordance with the anatomical substrates.

## 5 Primary nonaphasic alexia/agraphia

**Figure 3:**
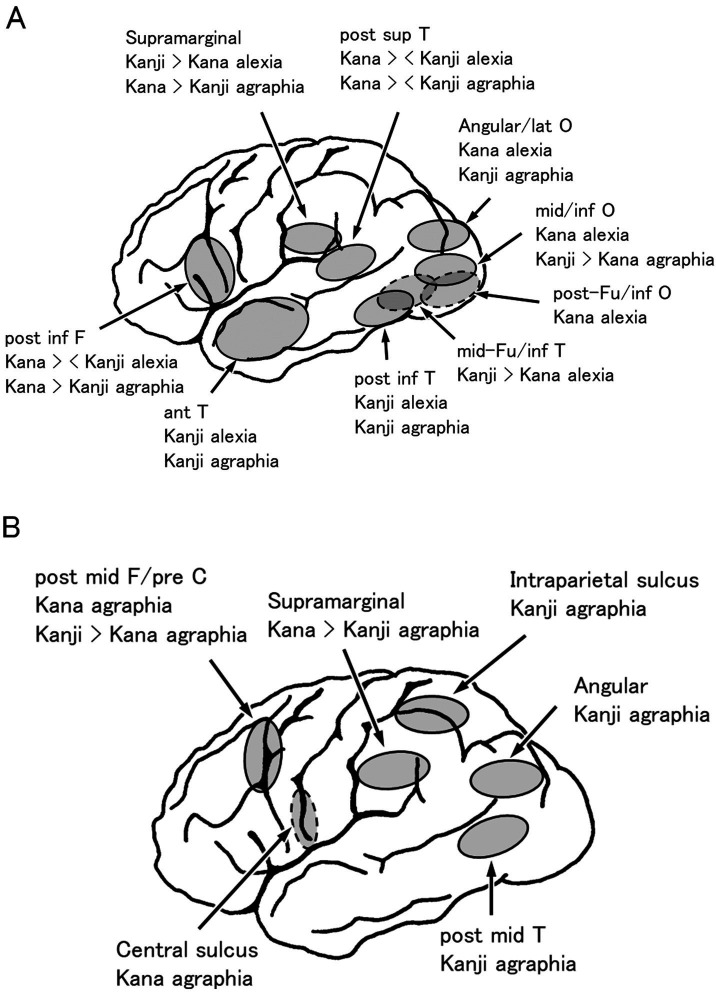
Alexia and agraphia in Japanese. **(A)** Lesion localization of pure alexia and aphasic/nonaphasic alexia with agraphia. Revised with permission from Yoshida et al. (2020). **(B)** Lesion localization of pure agraphia. Data are derived from our previous studies and Sasanuma (1975, 1980). Kanji > Kana: Kanji are more disturbed than Kana; Kana > Kanji: Kana are more disturbed than Kanji; Kana > < Kanji: Kana or Kanji are more disturbed.

Alexia/agraphia is divided into pure alexia (alexia without agraphia), alexia with agraphia, and pure agraphia (agraphia without alexia). Although “pure” means “only,” pure alexia is often accompanied by a minor degree of agraphia. The same also applies to “pure agraphia”: it is often accompanied by a minor degree of alexia. Figure 3 shows brain diagrams illustrating areas related to kana and kanji alexia and agraphia.

### 5.1 Pure alexia

Pure alexia is further divided into the splenium type or classical type, which involves the splenium of the corpus callosum, and nonsplenium type or nonclassical type (Kawamura, 1988).

#### 5.1.1 Splenium-type pure alexia

To produce alexia, not only the splenium, or forceps major, but also the left medial occipital gyri, or lateral geniculate body (Stommel et al., 1991), optic radiation (Maeshima et al., 2011), is necessary. Whether kanji or kana reading is more impaired depends on the extent of damage to the splenium and the involvement of the fusiform and lateral occipital gyri. In the previous reports of splenium type, kanji reading was more or no less impaired than kana reading (Fukunaga et al., 2010; Maeshima et al., 2011).

With regard to this point, the ventral part of the splenium receives projection fibers from the ventral V3 and V4 of the occipital lobe (Dougherty et al., 2005). Damage to this area can result in left hemialexia with <100-ms stimulus presentation and is more disturbed for kanji (Suzuki et al., 1998). If the splenium and posterior part of the callosal body are damaged, both kanji and kana cannot be read aloud in the left hemifield (Sugishita et al., 1986). Kanji-dominant recovery of reading 9 years later indicates that callosal fibers that transmit kanji visual information involve more extensive parts of the corpus callosum than kana does.

#### 5.1.2 Nonsplenium type: fusiform-type and posterior occipital-type pure alexia

Nonsplenium type is subdivided into the fusiform type and posterior occipital type. Previous multiple case studies identified the critical sites for pure alexia as the paraventricular white matter at the posterior horn (Damasio and Damasio, 1983), fusiform/lingual gyri (Isono et al., 1988), or ventral temporal cortices (Binder and Mohr, 1992). We reported a patient who exhibited pure alexia for the first time in the fusiform gyrus lesion after a first stroke in the lingual gyrus (Takada et al., 1998; Sakurai et al., 2000b). Subsequently, we reported a patient who had pure alexia predominantly for kanji in the mid-fusiform gyrus hemorrhage (Sakurai et al., 2006b). Most kanji reading errors had no response. The kanji reading performance was influenced by complexity, as measured by the number of stroke sequences, and imageability.

On the other hand, we encountered two patients with selective impairment of kana reading (Sakurai et al., 2001a; Sakurai et al., 2006b) caused by damage to the posterior occipital gyri. We later published an article on pure alexia for kana that summarized six patients, including the previously published ones (Sakurai et al., 2008b). The common lesion was in the posterior fusiform/inferior occipital gyri (Brodmann area [BA] 18/19), which coincides with the activation site in the covert reading of kana words (described later). Fusiform-type pure alexia and posterior occipital-type pure alexia have some features in common as well as differences (Table 5).

**Table 5:** Comparison of the fusiform-type and posterior occipital-type pure alexia.

Symptoms	Fusiform type	Posterior occipital type
Alexia	Kanji > kana^a^ Words > letters	Kana Letters
Impaired function	Whole-word reading	Letter identification
Kinesthetic reading	Kana words	Kana words
Lexical effects	Complexity, imageability (kanji)	Lexicality (kana)
Letter-by-letter reading	Kana nonwords	Kana nonwords
Word-length effect	Kana words	Kana words
Word-by-word reading^b^	Five-character kana words	None
Writing impairment	Kanji	Kanji
Visual discrimination	Normal	Mildly impaired

a Kanji reading is more impaired than kana reading.

b Compound words or sentences are read word by word or phrase by phrase.

Revised with permission from Sakurai et al. (2006b).

A single-character kanji is itself a word with multiple pronunciations, whereas a single-character kana corresponds to the Roman alphabet as a phonogram representing a single consonant–vowel unit or a phoneme. Thus, we proposed that pure alexia for kanji should be generalized to pure alexia for words, whereas pure alexia for kana should be generalized to pure alexia for letters (Sakurai et al., 2006b). From this point of view, in many previous studies of pure alexia, both fusiform and posterior occipital gyri lesions were reported, which we refer to as the “combined type” (Sakurai, 2004). In contrast, a few cases of fusiform-type pure alexia (Epelbaum et al., 2008; Morioka et al., 2011) and posterior occipital-type pure alexia (Caffarra, 1987; Mochizuki and Ohtomo, 1988; Uchiyama and Uchiyama, 1991) have been published.

### 5.2 Alexia with agraphia for kanji and pure agraphia for kanji

Iwata was the first to report alexia with agraphia for kanji or posterior inferior temporal (PIT)–type alexia with agraphia (Iwata, 1984, 1986). Subsequently, several case studies on the PITC lesion were published (Kawamura et al., 1987; Kawahata et al., 1988; Soma et al., 1989; Sakurai et al., 1994) and revealed the following features: (1) both single-character kanji and multiple-character kanji words were poorly read or written because of impaired character recall (Sakurai et al., 2000b; Sakurai, 2004), (2) agraphia was more profound and persisted longer than alexia did (Sakurai et al., 2000b), (3) kana reading was mildly impaired and showed letter-by-letter reading (Sakurai et al., 2000b), (4) kinesthetic reading was not always successful (Sakurai et al., 2000b), and (5) kanji reading was affected by complexity, frequency, and familiarity (Sakurai et al., 2006b), whereas errors in kanji writing were mostly due to impaired character recall (lexical agraphia), which was influenced by familiarity (Sakurai et al., 2008a).

Twenty years after the first report by Iwata (1984), similar cases of alexia with agraphia from a PITC lesion were reported in Western countries (Rapcsak and Beeson, 2004). In these cases, alexia and agraphia were more pronounced in irregular words, with coexistence of letter-by-letter reading. Because the essential deficit lies in impaired word recall, we designated this syndrome as “orthographic alexia with agraphia” (Sakurai, 2004).

**Figure 4:**
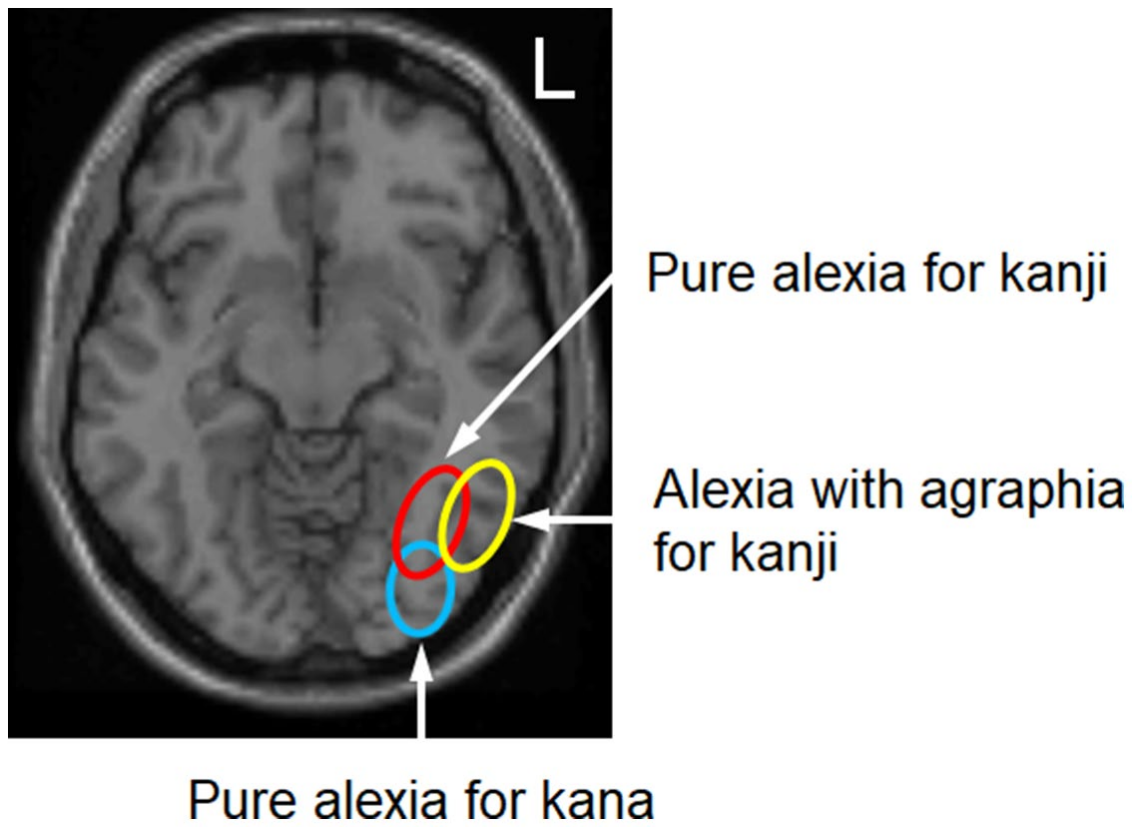
Location of the lesion of alexia with agraphia for kanji, pure alexia particularly for kanji, and pure alexia for kana. The lesion of alexia with agraphia for kanji is located in the mid-fusiform/posterior inferior temporal gyri (BA 37; yellow circle), whereas pure alexia, particularly for kanji, is located medially in the mid-fusiform gyrus (green circle). The lesion of pure alexia for kana is situated in the posterior fusiform/inferior occipital gyri (BA 18/19; blue circle). The brain single-subject slice template was reproduced from Statistical Parametric Mapping version 99 (https://www.fil.ion.ucl.ac.uk/spm/).

Two neuroanatomical problems have been noted (Sakurai, 2011). First, there are some similarities between PIT-type alexia with agraphia and fusiform-type pure alexia. That is, the PIT-type alexia with agraphia has letter-by-letter reading, as described above, whereas the fusiform-type pure alexia has kanji agraphia. The symptomatic distinction is that agraphia is more severe in alexia with agraphia for kanji and alexia is more severe in pure alexia for kanji (Sakurai et al., 2006b). Neuroanatomically, these two types have nearly the same lesion. The only difference is that the fusiform-type pure alexia has a lesion more medial to the PIT-type alexia with agraphia (Figure 4). Kawamura (1988) first indicated that the nonclassical (nonsplenium) type pure alexia is characterized by a lesion slightly more medial to the common lesion site of the PIT-type alexia with agraphia.

Second, in some individuals, alexia with agraphia for kanji resolved to pure agraphia for kanji in a similar lesion (Soma et al., 1989; Tsuruta et al., 1992; Komori et al., 2009). Furthermore, isolated agraphia for kanji occurred in nearly the same lesion (Yokota et al., 1990). Thus, some authors argued that alexia with agraphia for kanji proves to be pure agraphia for kanji or lexical agraphia in Western countries (Soma et al., 1989). We reported two patients with damage to the posterior part of the middle and inferior temporal gyri who exhibited pure agraphia for kanji (Sakurai et al., 2008a). Most of the writing errors consisted of no response due to impaired character recall. The kanji writing was influenced by character complexity, frequency, and familiarity. Taking these cases into consideration, we thought that alexia with agraphia for kanji and pure agraphia for kanji have separate neural substrates; that is, the lesion of pure agraphia for kanji is dorsolateral to that of alexia with agraphia for kanji in BA 37. This difference is consistent with the separate activation between reading and writing tasks observed in the imaging studies, which is described in the mechanism session.

#### 5.2.1 Accompanying common-name anomia

Alexia with agraphia for kanji is well known to be accompanied by common-name anomia (Soma et al., 1989; Sakurai et al., 1994). The anomia severity has been associated with the lesion that extends from the PITC to the anterior third of the temporal lobe or over the entire parahippocampal gyrus (Sakurai et al., 1994). Conversely, damage to the anterior third of the left temporal lobe was reported to cause common-name anomia and alexia with agraphia for kanji co-occurring with proper-name anomia (Sakurai and Ishizaka, 2025). We categorized proper-name anomia, common-name anomia, and alexia with agraphia for kanji (or orthographic alexia with agraphia) into the inferior longitudinal fasciculus syndrome because the PITC and the anterior temporal lobe are connected with the inferior longitudinal fasciculus.

#### 5.2.2 Two types of letter-by-letter reading

In pure alexia, letter-by-letter reading is frequently observed. Letter-by-letter reading is equivalent to pure alexia, with the exception of the co-occurrence of other reading or writing deficits (Behrmann et al., 1998). As previously described, letter-by-letter reading accompanies PIT-type alexia with agraphia (Sakurai et al., 2000b; Rapcsak and Beeson, 2004). We have reported another type of letter-by-letter reading, which we called “dorsal type” (Sakurai et al., 2020a). This type of letter-by-letter reading accompanies kana-predominant alexia and kanji-predominant agraphia. This pattern of alexia and agraphia is also observed in angular alexia with agraphia (described later). However, the angular gyrus was spared in this patient. The lesion was located subcortically in the middle and inferior occipital gyri (BA 19), situated intermediately between the angular gyrus and the posterior fusiform/inferior occipital gyri (lesion of pure alexia for kana) (Figure 3A).

The differences in dorsal-type letter-by-letter reading from conventional (ventral-type) letter-by-letter reading observed in pure alexia are that (1) kana or literal agraphia co-occurs and (2) kinesthetic reading is less helpful than that in pure alexia.

#### 5.2.3 Mechanism underlying alexia with agraphia and pure alexia

The lesion of alexia with agraphia for kanji, the PITC, or, more precisely, the mid-fusiform/PIT gyri (BA 37), is identical to the so-called visual word-form area (VWFA; peak activation, x = −39, y = −51, z = −18 in the Talairach coordinates) (Cohen et al., 2003) or the orthographic (input) lexicon, in which whole-word visual images of words and characters are stored as neural networks. In our positron emission tomography study, the mid-fusiform/posterior inferior temporal gyri were activated in the kanji word-reading task (peak activation, x = −44, y = −54, z = −22 in the Montreal Neurological Institute [MNI] coordinates) (Sakurai et al., 2000a). Damage to this area causes alexia with agraphia for kanji due to impaired recall of words and characters (Sakurai et al., 1994; Sakurai et al., 2000b) or, more generally, orthographic alexia with agraphia (Sakurai, 2004).

It should be noted that the peak activation locus of writing (i.e., the posterior middle/inferior temporal gyri [BA 37]) is 10 mm higher than the peak activation site of reading, that is, the mid-fusiform/PIT gyri or VWFA (orthographic lexicon): the peak activation was (x = − 42, y = − 54, z = − 12 in the MNI coordinates) (Beeson et al., 2003) for English word writing and (x = −52, y = −54, z = − 12 (Tokunaga et al., 1999); x = −43, y = −58, z = − 9 (Nakamura et al., 2002) in the MNI coordinates) for kanji word writing. Because the posterior middle/inferior temporal gyri are situated between the superior temporal gyrus (phonological lexicon) and PITC (orthographic lexicon, VWFA), damage to the posterior middle/inferior temporal gyri may interfere with the input of phonological information from the phonological lexicon to the orthographic lexicon or disrupt the visual image output from the orthographic lexicon to the parietal lobe. As a result, following damage to the posterior middle/posterior inferior temporal gyri, kanji or lexical agraphia (pure agraphia for kanji, as described earlier) may occur (Sakurai et al., 2008a) (Figure 3B).

Pure alexia particularly for kanji or pure alexia for words occurs when the visual information from the primary visual cortex to the PITC is interrupted in the mid-fusiform gyrus (Sakurai et al., 2006b), just medial to the PITC, involving the inferior longitudinal fasciculus. This type of alexia is accompanied by mild agraphia of kanji or lexical agraphia, likely because the lesion is close to the PITC (VWFA) and thus interferes with recall of the visual images of words and letters.

Pure alexia for kana or pure alexia for letters (Sakurai et al., 2001a; Sakurai et al., 2008b) is caused by impaired identification of letters in the posterior fusiform/inferior occipital gyri (BA 18/19), which is probably included in the anterior lateral occipital complex (LOC; specified for shape recognition) (Grill-Spector et al., 2001). In our positron emission tomography study, the posterior fusiform/inferior occipital gyri were specifically activated in the kana word-reading task (peak activation of reading aloud, x = −34, y = −92, z = −8 in the MNI coordinates) (Sakurai et al., 2000a, 2001c) and were found to be equal to the shared area of hypoperfusion across six patients with pure alexia for kana (Sakurai et al., 2008b).

#### 5.2.4 Anatomically constrained model of reading

Based on the results of our positron emission tomography (Sakurai et al., 2000a; Sakurai et al., 2001b; Sakurai et al., 2001c) and lesion studies, we modified Iwata’s model for Japanese (Iwata, 1984) and proposed a weighted dual-route hypothesis for reading that can also be applied to European languages.

**Figure 5:**
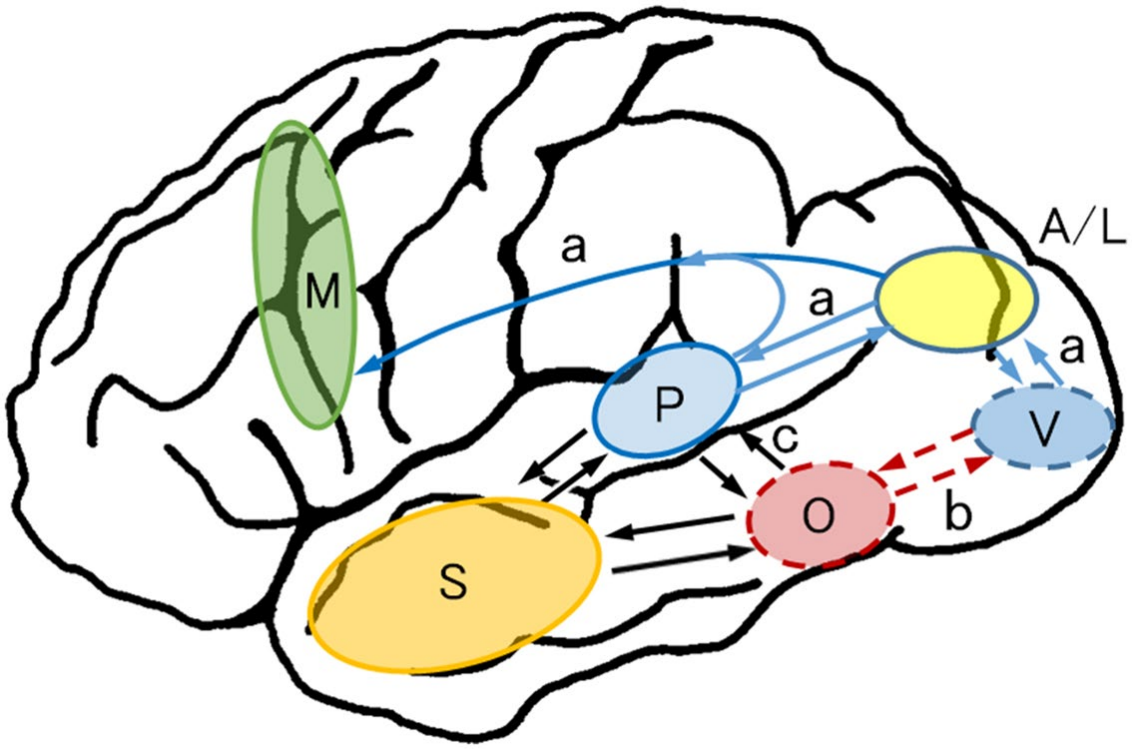
Anatomically constrained model of reading aloud: weighted dual-route hypothesis for reading. Reading requires phonological processing (a; grapheme–phoneme conversion) and orthographic processing (b; visual recognition of words). As visual images of words are stored in the orthographic lexicon (O), the orthographic route gains dominance in reading (weighted dual-route). a (blue), dorsal (phonological) route; b (red), ventral (orthographic) route; c (black), interaction between P, O, and S; A/L (yellow), angular/lateral occipital gyri, where grapheme–phoneme or phoneme–grapheme conversion is performed; M (blight green), motor and premotor area; O (red), orthographic lexicon (visual word form area; posterior inferior temporal cortex); P (blight blue), phonological lexicon (Wernicke’s area; posterior superior temporal gyrus); S (orange), semantic storage site; V (blue), primary visual cortex. Revised from Sakurai (2004).

Figure 5 shows the two main streams of information. The phonological (dorsal) pathway (a), which plays the role of successive phonological processing, proceeds from the primary visual cortex (V) to the posterior superior temporal gyrus (P) (phonological lexicon; Wernicke’s area) via the lateral occipital gyri (A/L: angular/middle and inferior occipital gyri) and the deep peri-Sylvian temporoparietal cortex (not depicted). The grapheme–phoneme conversion is performed in A/L. The orthographic pathway (b), which plays a role in the holistic recognition of words, proceeds from the V to the mid-fusiform/PIT gyri (O: orthographic lexicon; VWFA) directly or through the posterior fusiform/inferior occipital gyri. Reciprocal interactions exist among P, O, and the semantic storage site (S). The phonological pathway and orthographic pathway do not proceed in parallel: the orthographic pathway gains dominance in reading as visual images of words are stored in the VWFA (weighted dual-route hypothesis).

Kana or letter identification is disturbed in any type of alexia; thus, visual images of kana or letters are widely distributed in the occipital lobe and used for both phonology (grapheme–phoneme conversion) and orthography (whole-word recognition) (Sakurai, 2004). In contrast, kanji are recognized exclusively in the orthographic pathway (VWFA).

#### 5.2.5 Kinesthetic reading difficulty and tactually related cognitive impairments

Kinesthetic reading is a compensatory strategy for overcoming pure alexia. However, kinesthetic reading is less helpful in PIT-type alexia with agraphia (Sakurai et al., 2000b) and dorsal-type letter-by-letter reading (Sakurai et al., 2020a) (described earlier). On the other hand, difficulty in kinesthetic reading can occur with bilateral associative tactile agnosia and bilateral agraphesthesia (Sakurai et al., 2020b). Because these deficits share interrupted tactile or proprioceptive sensation from the hand and fingers, we refer to them as tactually related cognitive impairments (Table 6) (Sakurai et al., 2020b).

**Table 6:** Tactually related cognitive impairments.

Bilateral associative tactile agnosia
Bilateral agraphesthesia
Kinesthetic reading difficulty
Kinesthetic alexia (with eyes closed)
Visual-tactile discoordination^a^

a Impairment of visuotactually guided dexterity movement, for example, difficulty in equally dividing the flour mass (Sakurai, 2023).

The results of a functional imaging study showed that a subregion of the LOC called the lateral occipital tactile–visual (LOtv; peak activation, x = −47, y = −62, z = −10 in the Talairach coordinates) is activated in both visual and tactile naming (Amedi et al., 2002). In this regard, the two aforementioned patients with kinesthetic reading difficulty (Sakurai et al., 2020a; Sakurai et al., 2020b) had hypoperfusion of the LOtv (Sakurai, 2023). Based on this finding, we hypothesized that disconnection of the fibers from the parietal somatosensory area to the occipital LOtv or VWFA may cause difficulty in kinesthetic reading and other tactually related cognitive impairments (Sakurai et al., 2020b; Sakurai, 2023).

**Figure 6:**
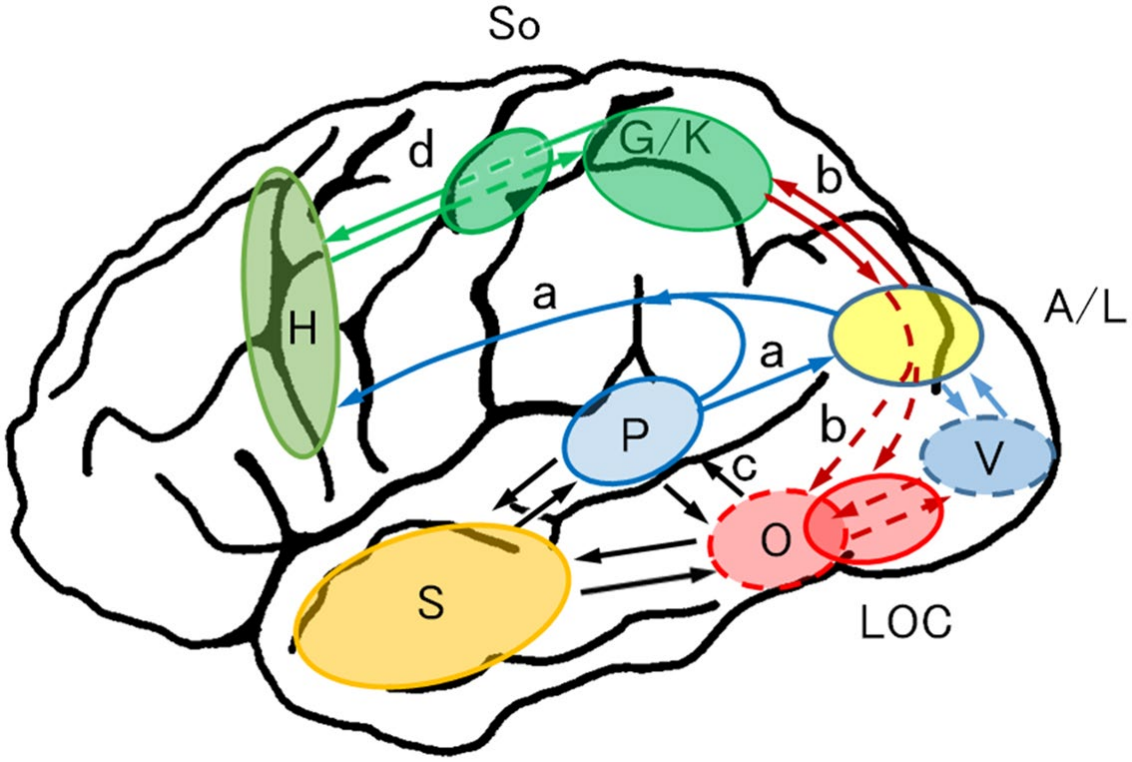
Anatomically constrained model of kinesthetic reading. The visual information of words and letters goes from the primary visual cortex (V) through the angular/lateral occipital gyri (A/L) to the graphemic/kinesthetic area (G/K) in the superior parietal lobule/intraparietal sulcus cortices where spatial and kinesthetic information of words and letters is stored (V → A/L → b → G/K). Visually linked kinesthetic information then proceeds to the motor and premotor hand area (H) to execute finger movement (G/K → d → H). Proprioceptive feedback from the finger movement goes upward to the contralateral (left) somatosensory cortex (So) and G/K area. The converted kinesthetic information is transmitted to the LOC and the orthographic lexicon (O; visual word form area), which enables successful kinesthetic reading. Graphesthesia uses the same feedback route (So → G/K → b → LOC, O). b (red), orthographic–kinesthetic reciprocal connection; G/K (green), graphemic/kinesthetic area, where visuokinesthetic and sequential motor engrams for words and letters are stored; H (blight green), motor and premotor hand area; LOC (red), lateral occipital complex; So (green), primary somatosensory cortex. Other abbreviations are denoted in Figure 5. Revised with permission from Sakurai et al. (2020b).

Figure 6 presents the main flow of information of kinesthetic reading and graphesthesia. In kinesthetic reading, the visual information of words and letters proceeds from the primary visual cortex (V) dorsally through the A/L to the graphemic/kinesthetic area (G/K; formerly the graphemic area; Sakurai et al., 2007, 2008a) in the superior parietal lobule/intraparietal sulcus (SPL/IPS) cortices, where the spatial and kinesthetic information of words and letters is stored (V → A/L → b → G/K). Visually linked kinesthetic information then proceeds to the motor and premotor hand area to execute finger movement (G/K → d → H). Proprioceptive feedback from the finger movement moves upward to the contralateral (left) somatosensory cortex (So) and G/K area. The appropriately converted kinesthetic information proceeds down to the LOC and the orthographic lexicon (O) or VWFA, which enables successful kinesthetic reading. Graphesthesia uses the same feedback route (So → G/K → b → LOC and O).

All tactually related cognitive impairments (Table 6) can be explained by damage to any of the dorsal kinesthetic routes (So → G/K → b → LOC → O). This model can also explain why tactile agnosia and agraphesthesia resulting from a parietal lobe lesion are unilateral (presenting in the contralateral hand), whereas those from an occipital lobe lesion are bilateral: the final pathway to lexical–semantic knowledge is in the left temporal lobe, and tactile information finally reaches the VWFA or visual object form area in the left temporal lobe whether the tactile input is from the left or right.

### 5.3 *On-kun* dissociation between semantic memory deficits and selective *on*-reading alexia

Patients who have progressive Gogi (word-meaning) aphasia, the Japanese equivalent of semantic variant primary progressive aphasia (as described earlier), exhibit anomia and surface dyslexia/agraphia. Characteristic errors are regularization (phonologically plausible errors, e.g., “pint” as “/pint/”) or legitimate alternative reading/spelling of components (Patterson et al., 1995; Weekes et al., 2003). Confusion between *on*-reading and *kun*-reading has been reported in Japanese patients with semantic dementia (Sasanuma and Monoi, 1975; Sakurai et al., 2006a; Sakurai et al., 2021), as follows:

1. Patients read a kanji character preferentially using *on*-reading (a kanji character is usually read with *kun*-reading, as described) and do not remember *kun*-reading (*on*-preceding and *kun*-deletion) (Sakurai et al., 2006a).
2. Two-character kanji word reading differs among *on*-reading, *kun*-reading, and specific-reading (*jukujikun*, irregular *kun*-reading) words: *kun*-reading is more impaired than *on*-reading is, and specific-reading is the most impaired (Sakurai et al., 2021).

In contrast to the above-mentioned *on*-reading predominance, we reported a woman with alexia with agraphia for kanji who exhibited selective impairment of *on*-reading after hemorrhage in the left posterior middle temporal gyrus (Yoshida et al., 2020). The patient read 98 single-character kanji with *kun*-reading at an accuracy of 98% (time: 5 min), which decreased to 71% (time: 23 min) with *on*-reading. She also correctly read *on*-reading words at 52%, *kun*-reading words at 87%, and specific-reading words at 82%. She tried to recall the *on*-reading by associating two-character kanji words containing the target character (e.g., 白 [shiro], *kun*-reading meaning “white” → 紅白 [kou-haku], *on–on* reading meaning “red and white” → 白 [haku], *on*-reading).

The *on*-reading predominance over *kun*-reading in semantic dementia and the reverse pattern in this *on*-reading alexia suggest double dissociation between the *on*-reading process and the *kun*-reading process. Given that *kun*-reading is directly linked to semantics, the *kun*-reading pathway corresponds to the lexical–semantic route (V2 → A2 in Figure 2; O → S → P in Figure 5), whereas the *on*-reading pathway corresponds to the direct pathway from the orthographic lexicon to the phonological lexicon ([a] in Figure 2; O → P in Figure 5). The patient with *on*-reading alexia may have damage to the direct pathway. The *on-kun* dissociation demonstrates the existence of two pathways—direct and indirect—from the orthographic lexicon to the phonological lexicon.

### 5.4 Alexia with agraphia and pure agraphia in the angular gyrus lesion

Dejerine (1891) first described alexia with agraphia due to an angular gyrus lesion. He proposed that the angular gyrus stored visual images of words and letters. However, recent lesion-to-symptom studies have implicated that PITC, not the angular gyrus, is the site for visual images of words (Sakurai et al., 2000b; Rapcsak and Beeson, 2004). It should also be noted that Dejerine’s original case had damage that extended to the lateral occipital gyri. Although the concept of “angular” alexia with agraphia seems established (Benson and Ardila, 1996), it remains unclear whether alexia with agraphia occurs in the circumscribed lesion of the angular gyrus (Yamadori, 1982).

In Western countries, it is known that damage to the angular gyrus causes lexical agraphia (Roeltgen and Heilman, 1984). In Japanese, angular alexia with agraphia presents with kana (phonological) alexia and kanji (lexical) agraphia (Kawamura, 1990; Miyake and Tanaka, 1992). We reported a patient with pure agraphia of kanji caused by a restricted infarction of the angular gyrus (Sakurai et al., 2000b) and a patient with alexia with agraphia (kana alexia and kanji agraphia) due to subcortical hemorrhage in the angular and lateral occipital gyri (Sakurai et al., 2010). Thus, damage to the lateral occipital gyri posterior to the angular gyrus may give rise to alexia symptoms in angular alexia with agraphia. In support of this view, a patient did not show alexia until he had a second infarction in the lateral occipital gyri in addition to the first infarction in the angular gyrus (Kuriyama et al., 2006). Kleist (1934) was the first to claim that alexia in the angular gyrus lesion results from extension of the lesion to the middle occipital gyrus, posterior to the angular gyrus.

#### 5.4.1 Angular-type alexia with agraphia

Kana alexia in angular alexia with agraphia is characterized by a deficit in letter identification and phonemic paralexia. The letter identification deficit involves letter-by-letter reading and the word-length effect, which are features of pure alexia. The observation that letter identification deficits occur not only in fusiform gyrus lesions (pure alexia) but also in lateral occipital gyri lesions (angular-type alexia with agraphia and dorsal-type letter-by-letter reading) suggests that the visual images of letters are diffusely distributed in the dorsal and ventral part of the occipital lobe and are used for both grapheme–phoneme conversion and whole-word recognition (described in the model of reading).

Phonemic paralexia mostly consists of substitutions of one or two kana characters (Sakurai et al., 2010). Transposition errors (changing the sequential order of two characters) are characteristic, for example, まなつ [manatsu], “midsummer” → なまつ [namatsu], nonword. This implies that the sequential phoneme-to-grapheme conversion is disturbed.

Although agraphia is marked for kanji in the angular-type alexia with agraphia, kana writing is mildly impaired (Kawamura, 1990; Miyake and Tanaka, 1992; Sakurai et al., 2010). It should be noted that errors comprise confusion between *hiragana* and *katakana*, in addition to phonological errors (phonemic paragraphia) and impaired character recall (Sakurai et al., 2010). The patient wrote *hiragana* words mixed with *katakana* characters (e.g., いきる [ikiku], “live,” H-H-H → イキる [ikiru], K–K-H; H, *hiragana*; K, *katakana*) and *katakana* words mixed with *hiragana* characters (e.g., ニワ [niwa], “garden,” K–K → にワ [niwa], H-K). These substitution errors are called allographic agraphia (see the brief description of the Japanese writing system), implying the presence of selection errors between allographs, and it is considered another type of phoneme–grapheme conversion disorder. Allographic conversion disorder typically results from damage to the parieto-occipital area, including the angular gyrus (Rapcsak and Beeson, 2002).

#### 5.4.2 Anatomically constrained model of spelling/writing of words

**Figure 7:**
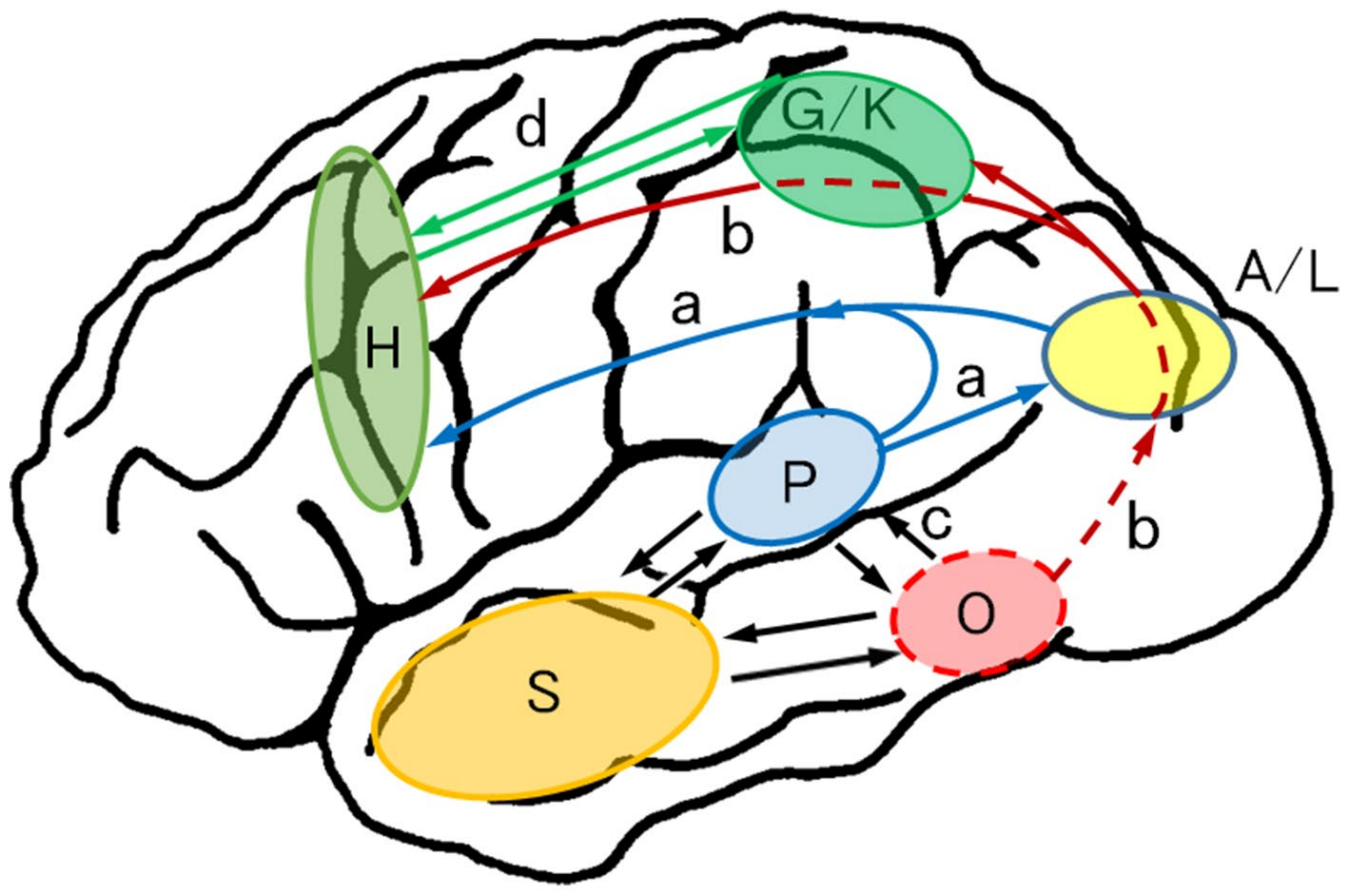
Anatomically constrained model of writing to dictation: the dual-route hypothesis for writing. Two main pathways are noted: the phonological pathway (P → a → H, or P → a → A/L → H) and the orthographic pathway (P → O → b → G/K → H). Phonologically recognized words in the phonological lexicon (P) go to the orthographic lexicon (O), where the phonological information is linked to the visual image of the word. On the other hand, phonologically recognized kana or syllable phonemes proceed to the angular gyrus and the adjoining lateral occipital gyri (A/L) to be converted to graphemes successively and enter the orthographic pathway. The orthographic pathway is divided into direct and indirect pathways in the parietal lobe. The direct pathway conveys holistic visual images of words and letters to the frontal motor and premotor hand area (H), whereas the indirect pathway enters the graphemic/kinesthetic area (G/K) in the SPL/IPS cortices where visuokinesthetic and sequential motor engrams for words and letters are stored and goes further to the hand area (H). b (red), orthographic pathway for writing. Other abbreviations are denoted in Figure 5. Revised from Sakurai et al. (2007).

Figure 7 presents the two main information flows in spelling/writing from dictation: the phonological pathway and the orthographic pathway. Phonological information proceeds from the primary auditory cortex (Heschl’s gyri) and the posterior superior temporal gyrus (P: phonological lexicon; Wernicke’s area) to the frontal motor and premotor hand area (H) along the arcuate fasciculus (a; phonological pathway) directly or through the A/L. Phoneme sequences of kana or a syllable are transmitted to the A/L, where the successive phoneme–grapheme conversion occurs and the visual information of kana joins the orthographic pathway (b).

The phonological information also proceeds from the superior temporal gyrus (P) to the PITC (O: orthographic lexicon; VWFA), where the phonological information is linked to whole-word forms. The phoneme-linked orthographic information then proceeds upward under the angular gyrus to the frontal motor and premotor hand area (H) (b: orthographic pathway). The orthographic pathway is divided into direct and indirect pathways in the parietal lobe. The direct pathway conveys the holistic visual images of words and letters to the frontal motor and premotor hand area (H), whereas the indirect pathway enters the G/K in the SPL/IPS cortices, where the visuokinesthetic and sequential motor engrams for words and letters are stored, and goes further to the frontal hand area (H).

The G/K in a broad sense includes, in addition to the SPL/IPS area, the inferior parietal lobule and parieto-occipital junction (superior occipital and precuneus gyri) and stores the visuospatial attributes of characters. Kanji characters that are graphically complex and have multiple-stroke sequences have a greater dependence on the orthographic route, whereas kana characters that link directly to phonemes and have a graphically simple configuration depend less on the orthographic route. The parietal G/K and the frontal hand area (H) have a reciprocal neural connection (d).

### 5.5 Kana agraphia/dyslexia in the supramarginal gyrus lesion

Kana agraphia or phonological agraphia occurs after damage to the supramarginal gyrus (Sakurai et al., 2010). Most errors are phonological (one or more substitutions in kana characters). In particular, transposition (changing the sequential order of two characters, e.g., ふしんせつ) ([hushinsetsu], “unkind” → ふんしせつ [hunshisetsu], nonword) was observed. This suggests disrupted sequential processing of characters in spelling/writing.

Some authors have reported selective or pure kana agraphia from the parietal lobe lesion including the supramarginal gyrus (Tanaka et al., 1987; Fujii et al., 1995). However, a detailed examination showed that kanji writing was mildly impaired due to defective character recall (Sakurai et al., 2010).

Kana agraphia is accompanied by mild to minimal phonological dyslexia of kana in the supramarginal gyrus lesion (Sakurai et al., 2010). In this case, similar to angular alexia with agraphia, transposition errors (i.e., changing the character sequences of the word) are observed. This indicates that sequential phonological processing of kana characters is performed from the angular/lateral occipital gyri to the supramarginal gyrus. It should be noted that in patients with damage to the supramarginal and angular gyri, verbal short-term memory is also impaired (Sakurai et al., 1998), which might affect the capacity for sequential processing.

Phonological dyslexia/agraphia and conduction aphasia share the same lesion of the supramarginal gyrus (Coslett, 2011; Ullrich and Roeltgen, 2011). In fact, these symptoms appear in the course of conduction aphasia (Alexander et al., 1992b; Beeson et al., 2010).

### 5.6 Apraxic agraphia

Apraxic agraphia is defined as (1) illegible grapheme formation that cannot be accounted for by sensorimotor dysfunction (Alexander et al., 1992a; Rapcsak and Beeson, 2002; Ullrich and Roeltgen, 2011), (2) improvement in grapheme formation with copying (Ullrich and Roeltgen, 2011), (3) preservation of oral spelling or typing (Ullrich and Roeltgen, 2011), and (4) disordered writing stroke sequences. We have added the fourth feature to these criteria (Sakurai et al., 2007). For example, the patient wrote a character as if he had drawn a line figure. Although the writing stroke sequence disorder is also observed in European languages (Coslett et al., 1986), it is easily observed in the Japanese kanji, which require complex sequential writing strokes.

The neural substrate for apraxic agraphia is located in the superior parietal lobule (Alexander et al., 1992a) or intraparietal sulcus area (Rapcsak and Beeson, 2002), where it is assumed visuokinesthetic and sequential motor engrams for letters and words are stored (G/K in Figure 7) (Sakurai et al., 2007). A meta-analysis of 18 imaging studies for handwriting identified an anterior SPL/IPS area (peak activation, x = −32, y = −38, z = 56 in the MNI coordinates) specific to handwriting (Planton et al., 2013). However, its role in handwriting is unknown. A lesion-to-symptom analysis suggests that anterior SPL/IPS damage causes disordered writing stroke sequences (Sakurai et al., 2007). In a broad sense, the G/K area includes more extensive cortices in the posterior SPL/IPS; inferior parietal, superior occipital, and precuneus gyri; and stores visuospatial attributes of characters (described in the anatomically constrained model of spelling/writing). This hypothesis might explain why some patients with apraxic agraphia exhibit deficits in letter imagery (impaired character recall) and severe deformity in writing characters (Crary and Heilman, 1988; Sakurai et al., 2007).

From a neuropsychological perspective, apraxic agraphia results from damage to the parietal G/K area per se (Sakurai et al., 2007) or disconnection of the output from the G/K area to the frontal hand area (H) (Otsuki et al., 1999). The disconnection type differs clinically from the cortical type in that disordered writing stroke sequences are more pronounced whereas grapheme deformity is minimal.

### 5.7 Frontal pure agraphia

Isolated agraphia is caused by damage to the left posterior middle frontal gyrus and surrounding cortices (Abe et al., 1993; Tohgi et al., 1995; Sakurai et al., 1997; Keller and Meister, 2014). This area has been traditionally called Exner’s area (Roux et al., 2010). Agraphia is characterized by kana or literal agraphia: letter substitution, omission, and perseveration are frequently observed. If the lesion involves the inferior frontal gyrus, deficits in kana or letter imagery (impaired character recall) occur (Sakurai et al., 1997). If the lesion involves the adjacent precentral gyrus, co-occurrence of apraxia of speech or kanji (or lexical) agraphia may result (Rapcsak et al., 1988; Saito et al., 1997; Sakurai et al., 1997; Kezuka et al., 1999).

The previously mentioned meta-analysis (Planton et al., 2013) revealed two separate frontal regions for handwriting: the posterior superior and middle frontal gyri (BA 6) specialized for handwriting and the inferior precentral/posterior inferior frontal gyri (BA 6) related to more general linguistic processing. The role of these areas is unknown. We consider these two areas to be functionally connected. That is, handwriting requires at least three sources of information (Sakurai et al., 2018): (1) visuokinesthetic information of a word or letter (the indirect orthographic route, V3 → V5 → V6 in Figure 2; O → (b) → G/K → (d) → H in Figure 7), (2) the whole-word graphic image that is converted from phonemes in the temporoparietal area (Sakurai et al., 2010) (the direct orthographic route, (d) in Figure 2; O → (b) → H in Figure 7), and (3) phonological information of the word transmitted from Wernicke’s area (the phonological route, A3 → A5 → (c) in Figure 2; P → (a) → H in Figure 7). These three information sources reach the premotor and motor hand area (H) and are linked to produce motor execution of words or letters. As described in the next section, the posterior superior/middle frontal gyri may receive visuokinesthetic and whole-word information, whereas the inferior precentral/posterior inferior frontal gyri may receive grapheme-linked phonological information,.

Some authors claimed that the phoneme-to-grapheme conversion was localized in the inferior precentral/posterior inferior frontal gyri (Omura et al., 2004; Ogura et al., 2013). However, it is more likely that the graphic images of characters are stored in the angular/lateral occipital gyri (described earlier) and that phoneme–grapheme conversion is performed in these cortices. What occurs in the motor and premotor area (H) is not conversion from phoneme to grapheme but rather a connection or integration of phoneme, grapheme, and visuokinesthetic information.

#### 5.7.1 Frontal phonological agraphia due to the inferior precentral gyrus lesion

As described above, kana or phonological agraphia occurs in the lesions of the frontal as well as parietal lobe. We reported a patient with phonological agraphia in a strict sense (i.e., writing impairment of five-character kana nonword only) (Sakurai et al., 2018). The agraphia was accompanied by disturbed mental arithmetic, which was assumed to be caused by reduced verbal short-term memory.

A subcortical hemorrhage in the left inferior (opercular portion) precentral gyrus was noted. This area is an endpoint of the arcuate fasciculus (Bernal and Altman, 2010), which may convey phonological information from Wernicke’s area via the supramarginal gyrus (a storage site for verbal short-term memory) (Sakurai et al., 1998). Thus, it is probable that the phonological route for spelling/writing terminates in the inferior precentral gyrus/posterior inferior frontal gyri and that damage to these gyri interferes with the linkage between the phonological information and the visuokinesthetic and whole-word information transmitted to the superior/middle frontal gyri from the parietal area.

#### 5.7.2 Frontal allographic agraphia

The inability to select appropriate letter shapes in spelling is referred to as allographic agraphia (Rapcsak and Beeson, 2002). In English, disturbed allographic conversion presents as confusion between uppercase and lowercase letters, whereas in Japanese, the confusion is between *hiragana* and *katakana*. The neural substrate of allographic agraphia is the parieto-occipito-temporal region (Rapcsak and Beeson, 2002) or the angular and lateral occipital gyri (Sakurai et al., 2010). Characteristically, the allographic errors are letter substitution in a word and unidirectional or bidirectional (upper- to lowercase and/or lower- to uppercase) conversion.

We reported a patient with behavioral variant frontotemporal dementia who exhibited allographic agraphia (Sakurai et al., 2024). This frontal allographic agraphia differed from parietal allographic agraphia in that we observed whole-word substitution as well as a few single-letter substitutions, and conversion was unidirectional from *hiragana* and kanji words to *katakana* words. Previous researchers reported a similar word-level substitution and unidirectional conversion to *katakana* in a patient with frontoparietal degeneration (Maeda and Shiraishi, 2018). The unidirectional conversion to *katakana* is partly explained by the compensation for the impaired recall of *hiragana* characters. However, the whole-word substitution may not be explained by perseveration only.

### 5.8 White matter pathways connecting modules

The inferior longitudinal fasciculus is a major network that connects the occipital lobe and the temporal lobe. The primary and association visual cortices (V in Figure 5) connect the orthographic lexicon (O) and further the anterior temporal semantic storage site (S) through the inferior longitudinal fasciculus. The phonological lexicon (P) and orthographic lexicon (O) have reciprocal connections via U-fibers. The middle longitudinal fasciculus connects the posterior superior temporal gyrus (P; Wernicke’s area; phonological lexicon) and the semantic storage site (S) (Makris et al., 2009), which is used for direct semantic access from Wernicke’s area.

The frontoparietal networks are composed of the superior longitudinal fasciculus (SLF). The SLF is divided into three parts: the dorsal branch that connects the superior parietal lobule and superior frontal gyrus (SLF I), the middle branch that connects the intraparietal sulcus area and superior and middle frontal gyri (SLF II), and the ventral branch that connects the inferior parietal lobule and inferior frontal gyrus (SLF III) (Parlatini et al., 2017). The input of phonological information to the Heschl’s gyri goes to the Wernicke’s area (P) and then enters the arcuate fasciculus/SLF III to the motor and premotor hand area (H). The LOC and the orthographic lexicon (O; VWFA) have a direct connection to the intraparietal sulcus area (G/K) via the vertical occipital fasciculus (Jitsuishi et al., 2020; Jitsuishi and Yamaguchi, 2020). The intraparietal G/K also has an interactive connection to the frontal motor and premotor hand area (H) through SLF I or II.

## 6 Secondary nonaphasic alexia/agraphia

Secondary alexia/agraphia is an impairment in reading or writing that is indirectly caused by other cortical or subcortical damage (Table 4).

### 6.1 Spatial agraphia and constructional agraphia

Spatial agraphia is the disturbance of graphic expression due to impaired visuospatial perception. It is characterized by (1) the addition of strokes, (2) slanted rather than horizontal lines of writing, (3) writing that occupies the right side of the paper, and (4) the insertion of blanks between a word (Hécaen and Albert, 1978). These features resemble writing impairment due to hemispatial neglect (Seki et al., 1998), which Hécaen and Albert (1978) acknowledged. Thus, in cases of hemispatial neglect, the concomitant agraphia may be called spatial agraphia.

Constructional agraphia is a writing disorder that assigns the spatial location of a word or a letter inappropriately. Errors comprise the omission of a part of a letter and an imbalanced spatial position and size of letters or words, which are referred to as dislocation and disproportion errors (Sakurai et al., 2007). These types of errors are marked in kanji and *katakana* (derived from a part of kanji) (Ota and Koyabu, 1970), which are graphically complex and require multiple-stroke sequences. Kleist first coined this term and thought it to be accompanied by constructional apraxia, which was attributed the left angular gyrus lesion (Kleist, 1934). However, as Kleist himself acknowledged, constructional apraxia does not always occur alongside constructional agraphia. The above illegible grapheme formation errors are also observed in apraxic agraphia. Thus, constructional agraphia may be a part of apraxic agraphia.

In the English literature, constructional agraphia is considered equivalent to spatial agraphia (Ullrich and Roeltgen, 2011). However, as described above, spatial agraphia results from a lesion in the right hemisphere (Ardila and Rosselli, 1993), whereas constructional agraphia may result from a lesion in the left parietal lobe. The problem here is whether it is appropriate to refer to deformed grapheme formation due to the right hemisphere as “spatial agraphia,” whereas deformation due to the left hemisphere is called “apraxic agraphia.” Because hemispatial neglect also occurs in the left hemisphere, we may designate deformed graphemes in the left hemisphere damage as “spatial agraphia.” Furthermore, if disproportion and dislocation deformities alone are observed in the left hemisphere damage, we may label this condition “constructional agraphia.” These types of deformity can occur when damage involves the posterior intraparietal area and the superior occipital gyri (G/K area in the broad sense in Figure 7).

### 6.2 Thalamic alexia/agraphia

Researchers have reported isolated alexia with agraphia or pure agraphia following damage to the thalamic nuclei. Both alexia with agraphia (Araki et al., 1990; Ito et al., 2022) and pure agraphia (Aiba et al., 1991; Ohno et al., 2000; Sakurai et al., 2011; Toyokura et al., 2011; Maeshima et al., 2012; Osawa et al., 2013) have similar lesions of mediodorsal and ventral lateral (VL)/ventroposterolateral (VPL) nuclei. Generally, there is greater impairment in reading or writing in kanji than in kana. Some patients with damage to VL/VPL nuclei exhibited poor grapheme formation (Sakurai et al., 2011; Toyokura et al., 2011; Osawa et al., 2013). Studies using single-photon emission computed tomography revealed hypoperfusion in the frontal or parietal lobe, suggesting diaschisis. The precise mechanism has recently been explained by the disconnection of association fibers between the thalamic nuclei and the connected cortical region (Katsuse et al., 2025).

We reported patients with damage to the VL/VPL nuclei who exhibited kanji agraphia with grapheme deformity and micrographia (i.e., a progressive reduction in character size during writing) (Sakurai et al., 2011). Poor grapheme formation and micrographia might be caused by a disruption of the cortico-subcortical motor circuit, which consists of the putamen, thalamus, premotor cortex, and sensorimotor cortex.

## 7 Conclusion

This article provided a review of the Japanese manifestations of alexia and agraphia. The features described above are not specific to Japanese but can be applied to all languages in general. The problem here is to clarify whether the same features of alexia or agraphia occur across countries. Further study is needed to elucidate the mechanism that underlies alexia and agraphia regardless of the differences in language.

This study’s limitation is the small number of patients on which the theory is based. To corroborate the hypothesis, a large number of patient studies are necessary.
